# Emergency care practitioners’ perceptions of the transition from student to independent practitioner: a qualitative exploration

**DOI:** 10.1016/j.afjem.2026.100959

**Published:** 2026-03-06

**Authors:** Dewan Lombard, Willem Stassen, Clint Hendrikse

**Affiliations:** Division of Emergency Medicine, Department of Family, Community and Emergency Care, University of Cape Town, Cape Town, South Africa

**Keywords:** Paramedics, Emergency medical services, Professional practice, Mentoring, South Africa

## Abstract

**Introduction:**

The World Health Organisation classifies emergency medical services (EMS) as an integral part of all well-functioning healthcare systems. The responsibility of making critical clinical decisions on a scene may be daunting to newly qualified emergency care practitioners (ECPs). The expectation that newly qualified ECPs must immediately function autonomously places considerable strain on their well-being, as undergraduate training alone may not equip them with the competencies and experience needed to practise with confidence. The aim of this study was to explore the views and perceptions of ECPs on their transition from student to independent practitioner, and the available resources in independent practice to support this transition.

**Methods:**

A qualitative exploration with semi-structured one-on-one online interviews was performed with ECPs across South Africa. The interviews were audio-recorded and transcribed verbatim. Interviews were analysed using content analysis with an inductive-dominant approach, grounded in constructivism. After data familiarisation was done, meaning units were condensed and led to the development of codes and categories.

**Results:**

A total of twelve ECPs were interviewed. Three categories were developed after the data analysis process: 1) new graduates feel under-prepared and overwhelmed, particularly when making high-risk clinical decisions and managing additional responsibilities, 2) feeling overwhelmed necessitates actions to change, such as seeking informal mentorship and developing coping strategies, and 3) there is a need for change in the workplace and university training, including a structured mentorship, better workplace support, and better preparation for paediatric and neonatal care.

**Conclusion:**

This study offers insights into the transition process from ECP student to independent practitioner, informing regulators on the necessary workplace support, mentorship structures, and curriculum adjustments for newly qualified practitioners to navigate their first months as independent practitioners.

## African Relevance

• Structured mentorship, internships and workplace support ease ECP transition from student to independent practice.
• Under-resourced EMS systems burden providers, erode confidence and compromise the quality and safety of patient care.
• Loss of experienced EMS staff threatens transfer of critical thinking and decision-making skills to newly qualified ECPs.
• ECP training tailored to African contexts and service realities should be prioritised to address graduate readiness gaps.

## Introduction

The World Health Organisation (WHO) classifies emergency medical services (EMS) as an integral part of all well-functioning healthcare systems, with EMS providers often serving as the first point of contact for patients in the pre-hospital setting [1]. The need for effective emergency care systems in Africa is particularly high due to the continent’s disproportionate burden of preventable and excess deaths [2].

In South Africa, newly graduated emergency care practitioners (ECPs) complete a four-year degree to become independent practitioners in the pre-hospital field. ECPs perform life-saving interventions for a variety of medical and trauma-related emergencies, including inter-facility transfers of the acutely ill, potentially distressing multi-casualty incidents, and are frequently subjected to hostile environments. The responsibility of making critical clinical decisions on a scene can be daunting to ECPs, who may have to navigate their first months working independently without supervision. This ‘transition shock’ from student to an independent practitioner may influence their resilience and self-efficacy to practise autonomously [3]. This alludes to a ‘theory-practice gap’ often accentuated as a contributor to the feeling of unpreparedness among ECPs as they are expected to commence independent practice [4].

Newly graduated ECPs partaking in critical care transports (CCTs) of patients with life-threatening conditions raise particular concerns about patient safety, outcomes, and the risk of adverse events, as critical care training is not standardised in the South African context [2,5]. Although ECP students complete clinical placement hours before independent practice, unlike most healthcare professions, ECP graduates do not have to complete any form of internship, mentorship, or additional training once they have graduated [4]. Emerging evidence indicates the need for robust educational programmes to ensure clinical competence and mental readiness after completing intensive training [6]. The expectation and responsibility placed on newly qualified ECPs to then function autonomously may also have a significant impact on practitioner welfare, as current undergraduate training alone may not provide the necessary competencies and experience for ECPs to practise with confidence.

The aim of this study was to explore the views and perceptions of ECPs on their transition from student to independent practitioner and the available resources in independent practice to support this transition.

## Methods

### Research paradigm and study design

An exploratory qualitative study utilising one-on-one online semi-structured interviews was used, grounded in a constructivist approach. This study relied on participants to construct meaning and understanding of their subjective views and perceptions, enabling the researcher to explore deep experiences and meanings through open-ended questions [7]. This study was approved by the Human Research Ethics Committee of the University of Cape Town (ref:133/2024).

### Setting

This study was conducted in South Africa and included ECPs from multiple provinces working in public and private sectors. As of September 2025, the Health Professions Council of South Africa (HPCSA) lists 1337 active ECPs concentrated in the Western Cape (*n* = 206) and Gauteng (*n* = 201); Limpopo, Mpumalanga, and the Northern Cape together have 22, and the Eastern Cape has 49 [8,9].

### Population and sampling

The study utilised purposive sampling followed by a snowball recruitment strategy. All practitioners registered as ECPs with the HPCSA with a minimum of one year of work experience post-qualification were eligible for inclusion. Participants not consenting to be interviewed or voice-recorded were excluded from the study. No eligible ECPs declined participation, and no participants withdrew from the study. The research team approached the initial four participants via LinkedIn and WhatsApp, as they could provide in-depth information based on their experience as established ECPs. After each interview, participants were invited to share the study information with other eligible ECPs, facilitating snowball sampling. Participation was diversified based on their area of service (public vs private) and the tertiary institution from which they graduated. A full description of the study, along with consent, was provided to all potential participants before scheduling the interview.

### Data collection instrument

An interview schedule was developed de novo by the research team, and a pilot interview was performed between DL and WS to maintain uniformity and the standard of interview technique. Data from the pilot interview was not included in the final analysis or results. The interview schedule contained four questions exploring the participant's experience as an ECP, their expectations prior to becoming an independent ECP, their management of difficult cases after graduating as an ECP, and their perspective on what changes are needed to help newly qualified ECPs transition from student to an independent practitioner. Pre-determined prompts were included for each question to support an in-depth exploration of the participants' views and perceptions.

### Data collection

Interviews were held online between the participants and DL (male) through Microsoft Teams (Microsoft Corporation, Redmond, USA) [10]. Interview dates and times were scheduled at the convenience of the participant and DL. Interviews were performed with audio recording and transcription enabled by DL, who was trained in qualitative interviewing. Field notes were not utilised in this study. No repeat interviews were conducted.

### Data analysis

Interview recordings were analysed using inductive-dominant content analysis according to the framework of Erlingsson and Brysiewicz [11]. Interviews were transcribed by the Microsoft Teams transcription service and manually verbatim thereafter by DL to ensure the correctness of the transcription. Data familiarisation was done by DL reading all the transcripts repeatedly, whereafter he identified meaning units related to the research aim. Our analysis focused on the manifest content of the interviews, what participants explicitly described, rather than deeper latent meanings or theory generation. These meaning units were condensed and labelled with codes, and categorised using NVivo 14 software (Lumivero Developers, Denver, USA) [12]. Codes were continuously compared within and across the transcripts, leading to the development of subcategories and categories. Data coding was performed by DL alone, followed by researcher triangulation, during which WS and CH independently reviewed the coding framework, categories, and representative participant quotations. Differences in interpretation were discussed and resolved by consensus, and the final coding framework was agreed upon by all authors.

### Reflexivity and trustworthiness

This study formed part of DL’s Master’s degree in Emergency Medicine at the University of Cape Town. DL worked as a flight paramedic for a company in helicopter and fixed-wing operations in the Middle East, while specialising in neonatal critical care, retrieval, and transport. As a novice qualitative researcher, DL was supported by WS and CH, who have extensive experience in qualitative research methodology and interviewing. DL articulated prior assumptions, particularly regarding neonatal care, and maintained a reflexive stance throughout the data collection and analysis.

Credibility was enhanced by using established research methods to perform data collection and analysis procedures [13]. DL employed neutral, non-leading questions while performing brief interview summaries to confirm the participant responses before progressing to the following questions. The researcher, a qualified ECP with over seven years of experience, brought contextual sensitivity from his personal transition from student to independent practitioner during probing.

Confirmability was promoted with DL, indicating predispositions [13]. The researcher recognised his own personal bias, relating to the shortcomings of neonatal critical care in the South African pre-hospital setting, which led him to avoid asking leading and ‘topic-focused’ neonatal questions actively. Initiation of neonatal topics came from the participants; however, DL asked direct follow-up questions relating to neonatal care at the end of the interview to explore recurring phenomena. DL noted an improvement in his interview throughout the data collection process as answers to the interview schedule came naturally from participants. WS and CH acted as secondary data analysts to further promote objectivity.

The dependability of this study was enhanced by providing a detailed description of the study design, supported by a coding tree, displayed in Table 1, describing how meaning units were abstracted into categories [13].

**Table 1:** Coding tree extract.

Meaning unit	Condensed meaning unit	Code	Subcategory	Category
*“I quickly moved into managerial and educational positions. Yet I still had to remain clinical”* P11	*“…quickly moved into managerial and educational positions…still had to remain clinical”*	Burden of clinical and leadership responsibilities	Perceived challenges test ECPs’ resilience	New graduates feel underprepared and overwhelmed
*“As time has gone on, obviously you learn how to deal with types of cases and patients, so that obviously helps”* P1	*“As time…gone on…learn how to deal…types of cases and patients…obviously helps”*	Repeated exposure leads to assurance	Establishing yourself as an ECP to build confidence and assurance	Feeling overwhelmed necessitates actions to change
*“I do believe in a form of mentorship. Every company needs mentors to help them employ newly graduated employees. They need to have a mentorship programme in place”* P5	*“…believe in…form of mentorship…every company needs mentors…to employ newly graduates…need to have a mentorship programme in place”*	Structured mentorship and life coaching	Workplace support programmes	There is a need for change in the workplace and university training

Transferability was promoted by providing in-depth and content-rich descriptions of the study context, which may enable readers to apply relatable findings to their specific context [13].

## Results

A total of twelve participants were included in the study. No participants withdrew from the study. Interviews, excluding the pilot, were held from June to September 2024. The interviewer was not acquainted with all the participants. Participants represented all four institutions in South Africa offering the BTech or BEMC programme, respectively. Participants had a diverse public and private sector employment background and a mean of 6.5 years of experience (range 3–16 years, SD 3.4). The duration of interviews ranged from 19 to 65 min (mean 42 min). Data saturation was confirmed after ten interviews, whereafter codes were formed and revised. An additional two interviews were conducted, and the absence of new codes confirmed data saturation [14]. A description of the participant details is presented in Table 2.

**Table 2:** Participant details.

Participant	Gender	Tertiary institution	Sector of Employment	Province	Experience
P1	Male	A	Public	Eastern Cape	6 years
P2	Male	B	Public and private	Gauteng	5 years
P3	Male	A	Public	Western Cape	6 years
P4	Female	A	Public	Eastern Cape	6 years
P5	Male	A	Private	Eastern Cape	3 years
P6	Female	B	Private	Gauteng	4 years
P7	Female	B	Private and Public	Gauteng	4 years
P8	Male	C	Private and Public	Western Cape and Eastern Cape	7 years
P9	Male	D	Private and Public	Gauteng and Northwest	10 years
P10	Male	D	Public and private	Limpopo	3 years
P11	Male	D	Private and Public	Northwest	16 years
P12	Male	C	Private	Western Cape	8 years

Institutions anonymised (A to D).

After data analysis, 559 codes were generated from the twelve interviews and inductively grouped into subcategories, ultimately forming three categories: 1) new graduates feel underprepared and overwhelmed, 2) feeling overwhelmed necessitates actions to change, and 3) there is a need for change in the workplace and university training. The categories and subcategories are included in Table 3. Additional quotes from participants are presented in a supplementary Table 4.

**Table 3:** Categories and subcategories.

Category	Subcategory
A. New graduates feel underprepared and overwhelmed	Perceived challenges test ECPs’ resilience
	Transitioning to independent practice is overwhelming
	University training may not be adequate to ensure readiness for real-world ECP responsibilities
B. Feeling overwhelmed necessitates actions to change	Developing self-preservation to prevent demotivation
	Establishing yourself as an ECP to build confidence and assurance
	Improvising on equipment shortages
	Psychological coping mechanisms to maintain mental well-being
	ECPs seeking mentor and self-established support
C. There is a need for change in the workplace and university training	Shortcomings to be addressed in university training
	Workplace support programmes

### A. New graduates feel underprepared and overwhelmed

This category explores the challenges and intricacies faced by newly qualified ECPs as they enter the workplace and independent practice. The following subcategories were developed:

*Perceived challenges test ECPs’ resilience*

Participants reported difficulties in balancing clinical and leadership responsibilities: *“quickly moved into managerial and educational positions…still had to remain clinical”* **(P11)***;* challenging workplace environments: *“you don't have a support system [in the workplace] to help you”* **(P4)***;* inexperience of doctors driving reliance on ECPs’ skills: *“they [doctors in the hospital] hear that you [the ECP] can do ABC&D, vastly being the intubation skills…and sometimes they [doctors] don't even try”* **(P1)**.

*Transitioning to independent practice is overwhelming*

ECPs reported mixed feelings of preparedness for independent practice: *“there's a lack of guidance in the transition and you definitely feel it”* **(P3)** and *“the feeling with babies is a lot more pressure”* **(P1)**; new ECPs describe the gap between expectations and reality of independent practice as ‘daunting’.

*University training may not be adequate to ensure readiness for real-world ECP responsibilities*

ECP university training was reported as inadequate for real-world responsibilities: *“they [university] don't really teach you those kinds of aspects [administrative responsibilities] at university”* **(P6)**; university curriculum being focussed on adult patient care: *“you spend 6 months with neonates compared to four years hands-on time with adults… your level of confidence is so much less [treating neonates]”* **(P6)**.

### B. Feeling overwhelmed necessitates actions to change

This category explores the workplace actions taken by ECPs to adapt to the challenges of independent practice, which often lead to feelings of underpreparedness and overwhelm among newly graduated ECPs. The following subcategories were developed:

*Developing self-preservation to prevent demotivation*

Reported as a sense of self-preservation: *“you need to fight to do your own job, but you are employed to do the job”* **(P4)** while being employed to fulfil the functions of an ECP.

*Establishing yourself as an ECP to build confidence and assurance*

Establishing yourself as an ECP happens with repeated exposure and leads to assurance; building confidence occurs over time: “guys [ECPs] in government level, you can see a huge difference [in their confidence and skillset] because in the private sector you're not really thrown in the deep end” **(P9)**.

*Improvising on equipment shortages*

Participants described how limited access to essential equipment necessitated improvisation through informal exchanges with colleagues across shifts, *“like a barter system”* **(P3)**. While not unique to newly qualified practitioners, these strategies emerged during transition as a means of navigating professional expectations despite systemic limitations.

*Psychological coping mechanisms to maintain mental well-being*

Coping involved personal evolution to maintain mental peace: *“by adapting the mindset of ‘I'm not God, I'm not there to save them, I'm there to be assistance”* **(P4)**; prioritising off-duty time, speaking to family members, and ruminating about the death of an infant formed part of ECPs' psychological coping mechanisms when facing difficult cases.

*ECPs seeking mentor and self-established support*

Mentor support during cases offered reassurance: *“just having someone to call makes a huge difference”* **(P5)**; participants described relying on self-established networks for support: *“if we needed any help, we actually called each other [peers]. There wasn't really a platform…to ask for help from a senior or practitioner”* **(P7)**.

### C. There is a need for change in the workplace and university training

This category describes the suggestions for change made by the participants to prevent newly graduated ECPs from feeling underprepared and overwhelmed during the transition from student to independent practice. The following subcategories were developed:

*Workplace support programmes*

Participants recommended workplace support programmes, including structured mentorship and internships: “I would recommend an internship programme. I feel as ECPs, after they qualify, at age 21 they are quite young. They don't have the experience of life, and they are given high risk responsibilities” **(P9)**; implementation of a workplace induction: “you have to spend maybe a week to work with someone, like just getting in touch and familiar with the environment you're going to be working in” **(P6)**.

*Shortcomings to be addressed in university training*

Refers to the knowledge, skills, and attitudes of newly graduated ECPs. Participants indicated that the current university B(EMC)/ BTech: EMC curriculum needs to be improved: “*university definitely did not prepare us well*” **(P7)**. Insufficient supported practice for high-risk procedures*: “the knowledge we get in university is good… transfer of that knowledge into practice is where the biggest gap is*” **(P12)**. ECPs need to practise humility, seek help, and be realistic about outcomes: “don’t be scared to consult” **(P11)**.

## Discussion

This study aimed to explore the views and perceptions of ECPs on their transition from student to an independent practitioner, and the available resources in independent practice to support this transition. We found that newly graduated ECPs entering independent practice feel underprepared and overwhelmed, necessitating them to adopt strategies to cope proactively. A need for change in the workplace and university training has been highlighted to avoid these complex feelings for future generations of newly graduated ECPs.

### New graduates feel underprepared and overwhelmed

Operational ECPs reported stepping into managerial and administrative duties (shifts, crews, and rosters) alongside clinical patient care -an expectation not well described in literature and possibly linked to staffing pressures and employer expectations of new graduates. This helps to explain why ‘transition shock’ persists among newly qualified practitioners despite clinical training. Reliance on newly qualified ECPs as student preceptors has been noted by Hanna et al. [6], who argue that to expect immediate post-qualification preceptorship is simply asking too much.

Participants perceived more clinical guidance in private EMS compared to public EMS systems. Heavy call volumes and understaffing in the public EMS system are likely to drive ECPs to rely on informal peer or mentor networks to navigate challenging environments [2]. Our data also suggest a sector contrast in perceived clinical guidance-an observation that, to our knowledge, has not been empirically compared locally and may warrant further investigation.

The causes for feeling overwhelmed and being under scrutiny in this study were consistent with other studies where the employer expects new ECPs to be job-ready after four years of university training [6]. ECPs have the highest level of pre-hospital emergency care education, which may elevate the expectancy of practice and level of care from EMS institutions. Newly qualified ECPs are often thrown into the deep end with little guidance. This correlates with Kennedy et al. [4], where ‘being thrown into the deep end’ may seem like a tradition or simply an excuse to accept ‘that is the way things are’. A lack of social integration could prevent newly qualified ECPs from experiencing a sense of belonging. Transitional anxiety is an avoidable process when students step into independent practice; however, this emphasises the need for support during this period, as supported by our study findings [3].

Training gaps were consistent with the literature reporting vast differences between university training and real-world responsibilities [6]. One area particularly emphasised was the limited time allocated to neonatal and paediatric preparation relative to adult training- consistent with South African studies - necessitating urgent reform of undergraduate education and system supports to improve patient safety [15].

### Feeling overwhelmed necessitates actions to change

With the transition from student to an independent practitioner, newly qualified ECPs are faced with the challenges of the workplace and the intricacies of practising autonomously. This forges a new journey of taking action to change or accept the challenges accompanying independent practice.

Participants reported becoming comfortable in their practice within six to twelve months, aligning with literature that the early post-graduation months are characterised by anxiety and low confidence, with consolidation over the first year [3]. Most participants relied on self-established support networks, making the transition survivable in low support contexts, for complex cases, unlike many global EMS systems, where clinical support is regulated and mandated [6]. Public-sector ECPs may develop greater confidence given the heavy, varied caseload characteristics of the public EMS system [2,16]. Although improvising due to resource constraints (barter system) may not appear specific to newly qualified ECPs, our participants framed it as an acute challenge early in practice — compounding the emotional burden of transition and highlighting the disconnect between training and operational realities. Similar networks of alternative communication and action to procure medical supplies have been reported in our local healthcare context [17].

### There is a need for change in the workplace and university training

A significant call for a formalised mentorship, internship, and life coaching programme for South African ECPs was consistent among participants, aligning with literature advocating for structured support and continuous feedback to ensure safe practice of high-risk interventions [18]. Clinical supervision can foster belonging, validation, and professional identity in newly qualified ECPs, many of whom are school leavers and young practitioners [3].

In Australia and New Zealand, paramedic education is a 3-year undergraduate degree with on-road clinical placement, followed by a 12-month internship year under a preceptor without the ability to practise independently [6]. Locally, perhaps, practitioners could register as ‘junior’ and ‘senior’ ECPs; senior status would require post-qualification experience, the ability to provide psychological safety, and demonstrated teaching capability for mentoring roles [19]. This avoids the model of the ‘everyone is a mentor’ which lacks depth in student education and could cultivate unwanted obligated feelings to be a mentor among experienced ECPs [6]. Several HICs have similar ranking orders in their EMS systems [4]. A structured workplace induction may help newly qualified ECPs orient to the system and find their feet [6].

A reconsideration of the current ECP training curriculum could include implementing rescue modules as a stand-alone entity and replacing them with clinical preparation. Addressing the shortcomings of the basic principles and concepts of administrative and managerial related skills in undergraduate training could be valuable; however, EMS institutions have different policies and regulations, making it impossible to have a blanket approach for all. The readiness of a graduate ECP to function autonomously depends on the individual’s ability to adopt and incorporate their organisation’s norms and values [4]. The introduction of an internship or mentorship programme would be more beneficial in addressing this issue, as mentors would be able to impart knowledge applicable to their specific system directly to the newly qualified ECP while providing clinical guidance and critical debriefing to help newly qualified ECPs bridge the gap between theory and practice. Reports of working alone are common among ECPs in our local context. Months-long pairing of an experienced ECP with a new graduate is likely impractical. Implementable options for a low-density ECP system could include: (i) limited early pairing for a few shifts and targeted scenarios, aligned with a preceptor framework that concentrates support in the early stages [19]; (ii) a centralised support desk staffed by a senior ECP for real-time advice; and (iii) virtual preceptorship including weekly case-based discussions to deliver supervision, feedback and peer support which has proven to be beneficial in resource-limited African EMS systems [20].

### Research gaps and implications for future work

Future research could evaluate structured mentorship or internship models for newly qualified ECPs in South Africa, including their impact on practitioner well-being, confidence, and patient care could help to address shortcomings in prehospital support structures. Possible differences between perceived support and exposure in public and private EMS settings could be identified to address inadequacies affecting ECPs across sectors. Finally, longitudinal research following ECPs over their first years of practice could provide insight into how professional identity, coping strategies, and support needs to evolve over time.

## Limitations

These results must be interpreted with the following limitations: A purposive sampling strategy was used to approach the first four participants from the researchers’ professional network; subsequent snowballing may have propagated the same social circles, introducing selection and access bias. Although data saturation was confirmed after twelve interviews, the final sample may still under-represent perspectives outside these networks. The study sample did not include all EMS systems and provinces in South Africa, and perspectives from underrepresented provinces, rural, and less resourced settings are limited. Male ECPs predominated in our sample. Readers should consider the differences between these contexts and their own when judging the transferability of the findings. Formal member checking of the preliminary categories was not undertaken. DL worked in the same capacity as some participants and held prior views on the topics discussed; however, residual observation and interpretation bias remain possible.

## Conclusion

The challenges accompanying the transition from student to an independent practitioner are complex. The responses of the participants echo the pleas for a formalised restructure of ECP training and workplace support that is needed to ensure the autonomy and sustainability of ECPs practising in South Africa. Safekeeping of our ECP workforce should be prioritised to ensure the transfer of critical thinking and decision-making abilities not attainable through theoretical knowledge from experienced ECPs to newly qualified practitioners. Future research should evaluate how the changes proposed by participants in this study could be implemented to assist tertiary institutions, health regulators, and EMS sectors in addressing the shortcomings experienced by newly qualified ECPs.

## Dissemination of results

Data have been shared with the HPCSA and tertiary institutions offering bachelor's degrees in emergency medical care in South Africa to inform them of the current challenges in the transition from student to an independent practitioner of newly qualified ECPs.

## Funding

Self-funded.

## CRediT authorship contribution statement

**Dewan Lombard:** Conceptualization, Methodology, Investigation, Data curation, Formal analysis, Validation, Visualization, Writing – original draft, Writing – review & editing, Project administration. **Willem Stassen:** Conceptualization, Methodology, Formal analysis, Validation, Writing – review & editing, Supervision. **Clint Hendrikse:** Conceptualization, Methodology, Formal analysis, Validation, Writing – review & editing, Supervision.

## Declaration of competing interest

WS and CH are editors of the journal, but were not involved in the editorial process or decisions around this submission. The authors declared no conflict of interest.

## Appendix Supplementary materials

Image, application 1
