# Supplementary material for: Emergency care practitioners’ perceptions of the transition from student to independent practitioner: a qualitative exploration
Source: Afr J Emerg Med. 2026 Mar 6;16(2):100959. doi: 10.1016/j.afjem.2026.100959 (PMC12993123; doi:10.1016/j.afjem.2026.100959)
Supplement: Supplementary file 1 [file mmc1.docx]

**Appendix. Supplementary materials**

Table 4 Additional quotes from participants

| **Category** | **Subcategory** | **Quotes** |
| --- | --- | --- |
| New graduates feel underprepared and overwhelmed | Perceived challenges test ECPs’ resilience | *“It's a new life, and you don’t want the new life not to have a life, you want them to stay on and live”* ***P1****.*  *“If I take where I'm working right now [private EMS], we have a system in place where we do case reviews after every case where there was anything weird about. That's not for punitive situations and punishment, and* *doesn't mean something went wrong”* ***P5****.*  *“I would say competition between fellow ECPs was a big thing. It's like everyone wants to prove themselves, and then there's very little teamwork and transparency”* ***P5****.*  *“The workplace expects that if you've now come out of university after four years, you are experienced, you know your stuff. You have what it takes to do the job”* ***P6****.*  *“I think that one of the barriers and probably the biggest barrier that a lot of newly qualified ECPs feel is that you don't want to feel you don't know what you're doing, you know?”* ***P12****.*  *“When I do approach people, I try not to come across as arrogant or anything like that due to the fact that we are ECPs and we're younger than all the people you're working with”* ***P5****.*  *“I believe if the workplace had support initially [when starting independent practice], things would have been better”* ***P10****.* |
|  | Transitioning to independent practice is overwhelming | *“As soon as I graduated, I actually had a lot of neonatal calls. I was not ready for that. It was highly stressful, and the courses that we did at university, no, that's not enough”* ***P7****.*  *“I would say in terms of the administration [managing rosters and crews as an ECP] part, I was not well prepared”* ***P6****.*  *“You know, there's no one [in independent practice] as like when you are a student looking over your shoulder, making sure everything is going well”* ***P1****.*  *“There was a gap in knowledge from what the university teaches us”* ***P4****.*  *“The fact that I had experience [4 years as NDip paramedic] made it way better in terms of me being confident [as newly graduated ECP] to applying my skills to patient treatment”* ***P8****.*  *“You are going to be on a response car, and every patient is going to be a P1. You will constantly be in an adrenaline rush. You are going to use all the fancy skills you learned, and then you get to the road, and the adrenaline rush, and P1 patient rarely happens”* ***P6****.*  *“Having had the previous 6-7 years’ experience before university, I kind of had an expectation because I worked with the paramedics before”* ***P3****.*  *“Many young people do not understand the responsibility of being a paramedic”* ***P3****.*  *“It sometimes helps if you have a friend to call, which I did. I had a doctor. He qualified and did his PhD, so he was the one holding my hand and showing me the ropes here and there. He was a mentor to me”* ***P10****.*  *“Unsure of myself, unsure if this is the job that I need to do. There were times when I felt: ‘Am I missing something?’ ‘Am I doing what I'm supposed to?’ ‘Is this the job for me?’ ‘Is it too late to change careers?’ This was especially the case when you encounter challenging situations where nothing you do helps the patient to survive”* ***P12****.*  *Once you qualify, you’re on the response vehicle by yourself, so you need to figure a lot of things out by yourself”* ***P10****.* |
|  | University training may not be adequate to ensure  readiness for real-world ECP responsibilities | *“I also had a target on my back due to some politics that happened in the varsity, so that is continuous, you know, proving myself, watching, having to keep my head high, doing*  *what I need to do, developing this kind of strong personality which I never had. On the practical side, it is a continuous fight to prove myself”* ***P5****.*  *“As soon as I graduated, I actually had a lot of neonatal calls. I was not ready for that. It was highly stressful, and the courses that we did at university, no, that's not enough”* ***P7****.*  *The degree itself was so taxing, and we [students] went through all that struggle and worked really hard to obtain our degree”* ***P7****.* |
| Feeling overwhelmed necessitates actions to change | Establishing yourself as an ECP to build confidence and assurance | *“I think one of the big things that I've realised was obviously that I needed to keep myself refreshed [with continuous medical developments]”* ***P12****.*  *As time passed by and you learn, and you progress to a better stage of knowing how to treat your patients better”* ***P4****.* |
|  | Psychological coping mechanisms to maintain mental well-being | *“When you just do a little bit of introspection and self-critique and discussion with peers and just try and be analytical about what you've been through and kind of take notes for yourself in terms of how you will do it differently next time”* ***P11****.*  *“You may have a lot of pressure on yourself in the sense where you almost feel like the responsibility is on your shoulders, especially when it comes to in-hospital patients, as they want you [ECPs] to do certain skills when the baby is not fully in your care as of yet”* ***P1****.* |
|  | ECPs seeking mentor and self-established support | *“Peers putting me on the side, saying: ‘Just breathe, you got it, you got it, you're doing well [advice from ILS peer colleagues on scenes]. I think that helped a lot”* ***P7****.*  *“A lot of it was having to figure it out for yourself. I would call my ex-practical partner. We used to work together for two years as students, and I’ll call him and ask for advice”* ***P5****.*  *“OK Google and EM guidance became my friend. If I didn't know what to do, I would call my university lecturer, who was very helpful in the first year. I called him a few times”* ***P4****.* |
| There is a need for change in the workplace and university training | Workplace support programmes | *“A mentor for me is one of the biggest things that you can have, especially when you start independent practice”* ***P8****.*  *“Look at other fields, look at medicine, a doctor has been studying for six years, and when he gets past the sixth year, he starts working a full-time internship. As an intern, I've noticed that people often ask, “Is that not what we should do with paramedics as well?”I feel it's something that has to happen”* ***P12****.*  *“There should be an acknowledgement that this is now a junior paramedic and the junior paramedic still needs to go through some supporting processes to the point where he can then get a change of label to a senior paramedic, meaning that he's been through all those aches and pains”* ***P11****.*  *“It's really difficult [to pair ECPs together on shifts] because of the scarcity of ECPs in South Africa. I understand that it's difficult to have the manpower to do that. But I feel that’s needed”* ***P7****.*  *“I do think that [mentorship] is lacking, and I've seen it with a lot of my peers that they don't have the confidence level because they were just thrown into the deep end”* ***P5****.*  *“My mentor’s role was just basically listening to clinical thoughts, which encouraged me and then made me more confident, and I've found ways to, you know, take a step back, figure out how to communicate, how to navigate through all of that mess [challenges of independent practice]”* ***P5****.*  *“[description of the ideal mentor] ECP with at least maybe 3-4, five years minimum, within that same system that this practitioner is going to come into. Because I think the big thing is always understanding the system. I think that's where a lot of the anxiety can be alleviated if you understand the system and what's required”* ***P12****.*  *“[description of the ideal mentor] I would say, someone who understands education, someone who is true, essentially has the people skills not to belittle someone else on the other end of the line. I feel like it can't just be, "Here's the list, guys." You're going to be on this week. I'll be on that week. It needs to be someone who is in that role for clinical guidance and debriefing”* ***P2****.*  *“I think a minimum of six months [duration of mentorship programme]”* ***P8****.*  *“I think how long you need to be supported for would be very individual-based”* ***P2****.*  *“I would definitely tell any new graduate to be careful of burnout”* ***P11****.*  *“Have a good psychologist”* ***P5****.*  *“I think the most important thing is to remain humble. Because humility breeds success, and it will take you a long way”* ***P9****.*  *“Do not think you're going just to save lives. You need to come back to reality because, when you step into a workplace as a new ECP, you are not living in the real world. The university makes you think you'll be saving lives the whole time. They are not telling you how many people will die. There is a need to be realistic”* ***P4****.*  *“Throughout your career, there needs to be a lot more constant training”* ***P1****.*  *“Make them [students] aware that CMEs are important”* ***P7****.*  *“I would tell them the importance of learning to know your system. The system in which you work”* ***P11****.*  *“In South Africa, with the HPCSA, the following up of adversary actions or adverse clinical outcomes is very minimal, and unless reported by the patient and the patient’s family”* ***P3****.* |
|  | Shortcomings to be addressed in university training and the workplace | *“There's a whole debate regarding Rescue forming part of the EMC curriculum. I think Rescue should stand on its own. They can add more paediatrics and neonates”* ***P9****.*  *“They [university] could have focused more on theory for us on the clinical treatment of patients and less on rescue because, in the past 6 years’ experience, I think I've done about four rescues”* ***P4****.*  *“I do feel what contributed [to a successful university programme] was it basically being half previously qualified or short course guys and half of the students from school which complemented it, in bringing some guys that hadn't done EMS then joined the university that had basically life experience that I feel what we had done in university is very much what the model should look like”* ***P3****.*  *“I would say it's not to walk in coming as the big fish in the small pond, if that makes sense”* ***P2****.*  *“It doesn't matter if the person's coming from a basic life support background. You will always learn something from anyone. So be open-minded and improve yourself”* ***P7****.*  *“Don't be scared to consult. And there we can learn a lot from the doctors. The doctors are being groomed from the get-go into knowing that I am the general practitioner or community service doctor, but in my system, I've got the specialist now [to consult]. Unfortunately, in EMS, we don't have these specialists”* ***P11****.*  *You [universities] need to give the neonatal and paediatrics module a year on its own, I think, although we touch on it, it just gave us the basics”* ***P9****.* |
